# An automated and objective cover test to measure heterophoria

**DOI:** 10.1371/journal.pone.0206674

**Published:** 2018-11-01

**Authors:** Clara Mestre, Carles Otero, Fernando Díaz-Doutón, Josselin Gautier, Jaume Pujol

**Affiliations:** 1 Davalor Research Center (dRC), Universitat Politècnica de Catalunya, Terrassa, Spain; 2 Centre for Sensors, Instruments and Systems Development (CD6), Universitat Politècnica de Catalunya, Terrassa, Spain; 3 Inria, Sophia Antipolis, France; Justus Liebig Universitat Giessen, GERMANY

## Abstract

Heterophoria is the relative deviation of the eyes in absence of fusional vergence. Fusional vergence can be deprived by, for example, occluding one eye while the other fixates a visual target. Then, the occluded eye will presumably deviate from its initial position by an amount that corresponds to the heterophoria. Its assessment in clinical practice is crucial for the diagnosis of non-strabismic binocular dysfunctions such as convergence insufficiency. Traditional clinical methods, like the cover test or the modified Thorington test, suffer from practitioner’s subjectivity, impossibility to observe the occluding eye or unusual viewing conditions. These limitations could be overcome by using eye tracking systems to measure objectively the heterophoria. The main purpose of this study was to compare the performance of an automated and objective method to measure near heterophoria using an eye-tracker with two conventional methods: the cover-uncover test and the modified Thorington test. The eye tracking method gave us the possibility to measure the heterophoria as the deviation of the occluded eye (mimicking the cover test) or as the deviations of the occluded and fixating eyes (adhering to the theoretical definition of heterophoria). The latter method provided smaller results than the former, although on average the differences might not be clinically relevant. The proposed objective method exhibited considerably better repeatability than the two conventional clinical methods. It showed better agreement with the modified Thorington test than with the cover-uncover test, and a similar level of agreement was obtained between the two clinical methods. To conclude, the use of eye-trackers to measure heterophoria provides objective and more repeatable measures. As eye-trackers become common tools in clinical settings, their use to measure heterophoria should be the new gold standard.

## Introduction

A normal functioning of binocular vision, including both sensory and motor components, guarantees proper alignment of the eyes. While the sensory fusion component unifies the perception of the images of the two eyes, the motor fusion component is responsible to align the eyes in such a manner that sensory fusion can be maintained. If one eye is artificially excluded from participating in vision (i.e., the sensory and motor fusion components of binocular vision are suspended), a relative deviation of the visual axes may appear in most subjects, which is called heterophoria [1]. When the fusion mechanism does not function properly, a manifest deviation of one eye is present. This deviation is called heterotropia, or strabismus. While heterotropia is a manifest deviation, heterophoria is latent and becomes evident only when the normal fusion mechanisms are disrupted. This deviation may be horizontal, if the visual axis of one eye converges or diverges more than the other; vertical, if one visual axis is higher than the other; or cyclorotary, if there is a misalignment of the eyes due to a clockwise or counterclockwise rotation of one eye.

Since an abnormal value of heterophoria may lead to symptoms like visual fatigue, headache or double vision [2], it is routinely assessed in clinical optometric practice. There are several methods to measure heterophoria such as the cover test or the modified Thorington test.

There are different variants of the cover test. The unilateral cover test consists in covering one eye and observing the movements of the other eye. If the non-occluded eye moves to take up fixation, the patient exhibits heterotropia. If contrarily there is no movement of the fellow eye, that eye is then covered and the other eye is observed. Once it has been established that the fellow eye does not move when either eye is covered, a cover-uncover test is typically performed to determine whether the patient has a heterophoria. The cover-uncover test is equivalent to the unilateral cover test but now the examiner observes the movements of the occluded eye when the cover is removed. If heterophoria is present, the covered eye moves to its heterophoric position and when uncovered, the eye makes a movement in the opposite direction to recover fixation. The alternate cover test brings out the maximal ocular deviation regardless of whether it is a heterophoria or heterotropia. In this case, the occluder is quickly switched from on eye to the other avoiding any period of binocular fixation between occlusions. In the three variants of the cover test, the deviation can be measured with a prism bar as the amount of prism diopters (PD) needed to cancel out the recovery (or re-fixation) movement (prism cover test). A prism is defined as having 1 PD when it causes a deflection of a light ray of 1 cm measured at a distance of 1 m. Thus, the degrees of eye rotation can be transformed into PD as 100 times the tangent of the rotation angle. The cover test is considered an objective method since the result does not depend on the answers of the patients, although it depends on the criteria and ability of the examiner [3–5].

The modified Thorington test is a subjective method that uses the Bernell Muscle Imbalance Measure (MIM) card (Bernell Corp., Indiana) to measure heterophoria. It has a row and a column of numbers that are separated by 1 PD at 40 cm. A penlight is shown to the patients through a hole in the center of the card while they hold a Maddox rod before the right eye. Patients are asked through which number the line created by the Maddox rod passes and on which side of the penlight’s light.

Several studies concluded that the different tests to measure heterophoria are not interchangeable due to their low level of agreement [6,7]. There is controversy about the most repeatable test, but it is typically agreed that the cover test and the modified Thorington test offer the best results in terms of repeatability [7–9].

The use of the cover test to measure heterophoria in clinical practice is extremely common. However, it suffers from several limitations such as its non-objectivity. Although the result does not depend on the answer of the patients, it depends on the examiner. Several authors have found no clinically relevant mean differences between experienced and novice examiners, although the 95% limits of agreement were rather wide [4,5]. Another source of interexaminer variability might be the use of a different criterion for the neutralization point [3]. The endpoint of the movement that should be recorded as the result of the test is still unclear. One possible endpoint is the first amount of prism with which no movement is seen (first neutral). Other possibilities are any point in the range of prism after the first neutral in which no additional movement of the eye is seen, or the prism that causes an opposite movement of the eye (reversal point) [3]. It is generally accepted that some execution aspects such as the time of occlusion have a direct influence on the measured heterophoria [4,10]. The poor resolution is also a limitation of the cover test. Several authors showed that under ideal conditions, the smallest eye movement that a person (the examiner) can detect with unaided eye is 2 PD [11,12]. As a consequence, the threshold commonly used to decide whether differences are clinically significant is 2 PD. This value has not been established on the grounds of diagnostic significance but based on a limitation of the measurement test. Finally, the fact that basically the covered eye cannot be observed represents an impediment to analyze how the eye reaches its heterophoric position [13].

It is generally accepted that the modified Thorington test is simple and easy for patients to understand. However, its principal drawback is its subjectivity, as the results solely rely on the answer of the patients. A specific emphasis needs to be placed on asking the patients to keep the grid and numbers of the MIM card focused. The dissociation system used, which creates rivalry between the eyes and unusual viewing conditions, does not favor a proper control of accommodation.

These limitations could be overcome by using eye tracking systems. The calculation of heterophoria from the eye-tracker’s recordings relies solely on patient actual eye deviation, and not his subjectivity nor the one of the examiner. Moreover, if the proper occluder is used, it becomes possible to register the movements of the covered eye and automate the whole process in order that the test is always executed equally. The first studies using objective eye tracking systems during the cover test described the dynamics of eye movements during both the cover and recovery phases [10,14]. More recently, several works used different eye tracking techniques to measure heterophoria and compared the results with clinical methods. Han et al. used a limbus eye tracking system and an haploscope to quantify objectively heterophoria. They obtained a precision from 0.7 PD to 1.1 PD, similar to the Maddox rod method and the alternate cover test, but a resolution of 0.17 PD, which is noticeably better [15]. Moreover, a strong and significant correlation between the phoria measured with the limbus eye tracking system and the Maddox rod method (*r* = 0.85, *p* = 0.008) was observed. In the version of the Maddox rod method that Han et al. performed, subjects viewed a penlight in primary position. Their right eye was occluded for 15 s and then it was uncovered and covered rapidly to assess the position of the red line on a calibrated grid. This was repeated until the subjects could report on which number the red line appeared. Babinsky et al. used the MCS PowerRefractor (Multi Channel Systems, Reutlingen, Germany) to assess simultaneously accommodation and ocular alignment data in children [16].

Other studies used eye tracking systems to obtain objective measurements of other binocular vision parameters, such as fixation disparity [17–21]. Fixation disparity is a small vergence error common even among subjects with normal binocular vision. It can be measured subjectively with psychophysical methods or objectively using eye-trackers. These studies found significant correlation between the objective fixation disparity and heterophoria. Additionally, Svede et al showed that objective fixation disparity is different depending on whether the calibration is performed in monocular or binocular vision and found no correlation between fixation disparity and heterophoria when the eye-tracker is calibrated binocularly [21].

In the current study, the eye-tracker EyeLink 1000 Plus (SR Research Ltd., Ontario, Canada) was used to record eye movements during the performance of the cover test at near distance. Two different methods, detailed in subsequent sections, were used to measure heterophoria from the eye-tracker’s recordings. In the first method, the deviation of only the occluded eye from its previous binocular position was considered in order to measure heterophoria, as it is done when the conventional cover test is performed in clinics. However, there is evidence that the fixating eye might occasionally move in the same direction as the covered eye instead of remaining still as it is generally assumed [22]. Since by definition heterophoria is a relative deviation between the two eyes, the deviation measured with the prism bar might in fact be greater than the true heterophoria. Thus, in the second proposed method the deviations of both the occluded and fixating eyes from their previous binocular positions were considered to compute it. The main objective of this study was to determine the differences between these two methods of heterophoria computation and validate them with the cover-uncover test and the modified Thorington test.

Pickwell stated that the fixating eye often moves, particularly during the recovery phase (i.e. when the occluder is removed) and observed that the amplitude of this movement is greater when the dominant eye is covered [23]. Peli et al also noted similar movements of the fixating eye during the recovery phase and obtained significantly different responses between the two eyes, especially for those subjects with clear ocular dominance [14]. The symmetry of heterophoria between the two eyes and the effect of motor ocular dominance was analyzed in the current study. It is hypothesized that the greater amplitude of the fixating eye’s movement when the dominant one is occluded might be justified by a greater deviation of the dominant eye (greater magnitude of heterophoria).

## Methods

### Subjects

Thirty non-presbyopic adults (15 females and 15 males) participated in the study. Their mean age ± standard deviation (SD) was 27.9 ± 4.6 years and ranged from 21 to 38 years. All had 20/25 or better visual acuity in each eye at far and near distance with their habitual correction and no manifest deviation (strabismus) as measured with the unilateral cover test. The horizontal heterophoria at near assessed with the cover-uncover test and measured with a prism bar ranged from +14 PD (esophoria) to -14 PD (exophoria).

All subjects were informed about the nature of the study before starting the experimental procedure and signed informed consent. The study followed the tenets of the Declaration of Helsinki and was approved by the Ethics Committee of Hospital Mutua de Terrassa (Terrassa, Spain).

### Materials and instrumentation

Heterophoria was measured with three different methods: the cover-uncover test with a prism bar, the modified Thorington test and the automated and objective cover test using an eye-tracker.

During the cover-uncover test patients were asked to hold a visual acuity card at 40 cm and fixate a 20/30 visual acuity letter. The examiner covered the patients’ eyes with an opaque occluder and a prism bar with powers of 1, 2, 4 to 20 PD in 2 PD steps and powers of 25 to 45 PD in 5 PD steps (Gulden Ophthalmics, Elkin Park, PA, USA) was used to measure horizontal heterophoria.

The horizontal heterophoria was also measured with the modified Thorington test using a Maddox rod and the MIM card. It has a measurement range from 28 PD esophoria to 28 PD exophoria with a resolution of 1 PD.

**Fig 1:**
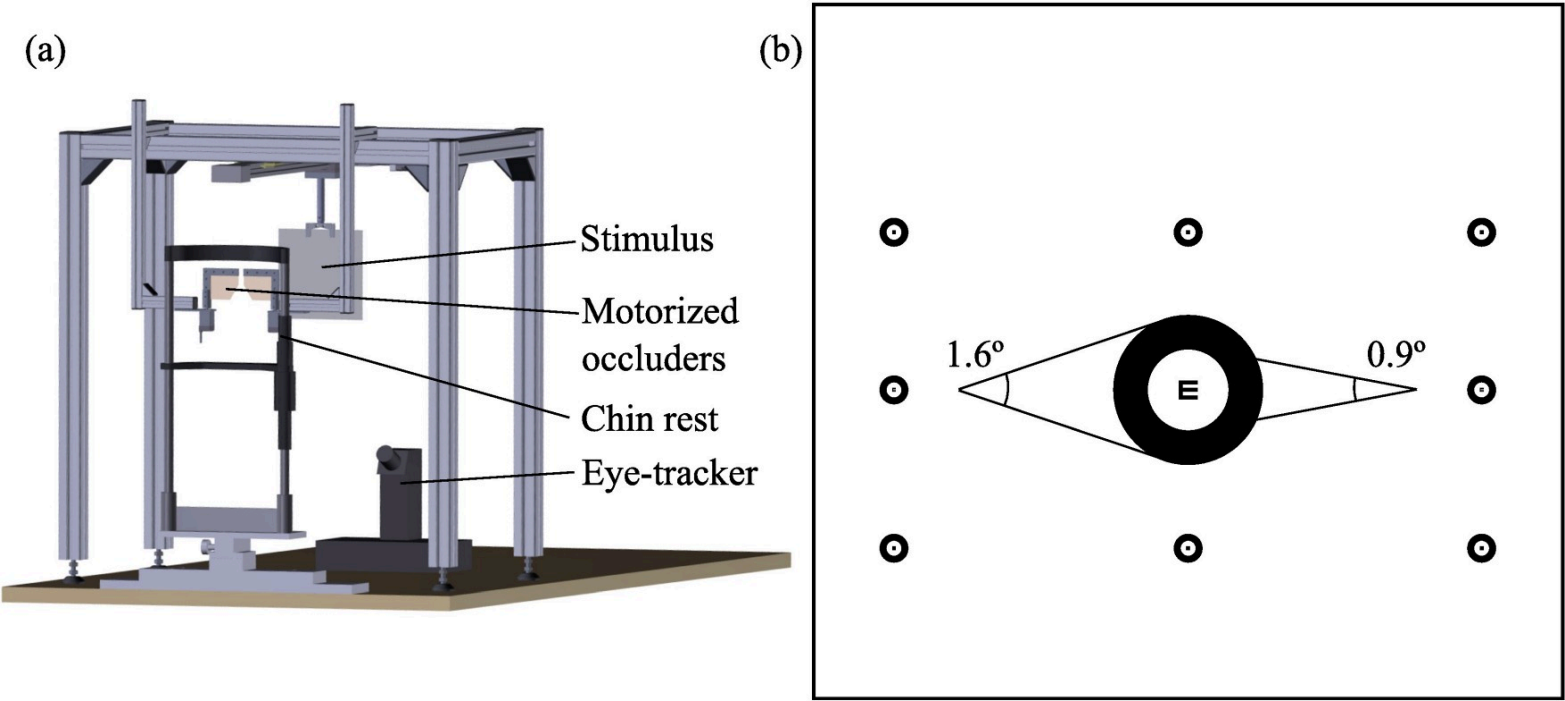
Instrumentation and visual stimulus. (a) Schematic representation of the experimental setup. (b) Visual stimulus. The central target has been enlarged for the sake of visibility. The other eight stimuli were identical and used to calibrate the eye-tracker.

Finally, heterophoria was assessed objectively with an infrared video-based eye-tracker. Binocular eye data were registered with an EyeLink 1000 Plus at a sampling rate of 250 Hz (Fig 1A). The visual stimulus was printed on a white card which covered a visual field of 40.5° x 42.9° and placed at 40 cm of the patient. The fixation stimulus consisted of an empty black circle of 1.6°. The inner white region subtended an angle of 0.9° with a 20/50 (0.21°) Snellen E letter at the center to favor fine fixation and proper stimulation of accommodation (Fig 1B). The fixation stimulus was placed 16° downwards from primary position in order to optimize eye-tracker’s data quality. Primary position refers to the position assumed by the eye when one is looking straight ahead with body and head erect. All eye movements were within the gaze tracking range reported by the manufacturer of 32° horizontally and 25° vertically. While patients fixated the Snellen E they also viewed peripherally the other eight identical stimuli at an eccentricity of 16° horizontally and 9° vertically used to calibrate the eye-tracker. The subjects’ head was restrained using a chin rest.

In order to disrupt fusional vergence, each eye was occluded in turn similarly to the cover-uncover test. The occluders consisted of two crossed polarizers which blocked visible light but transmitted infrared wavelengths, hence allowing to register eye movements continuously even when the eyes were occluded. In order to check that the two pairs of crossed polarizers completely occlude the fixation target, their transmittance was measured with the spectrometer SPECTRO 320 (Instrument Systems, GmbH, Germany). Their mean transmittance in the visible range (from 380 nm to 780 nm) was 0.63%. It was also verified by visual inspection that observers could not have a residual view of the target through the occluders. They were driven by two stepper motors and controlled with a custom software coded in Matlab R2017a (MathWorks, Natick, MA, USA). It took approximately 0.27 seconds to occlude completely the visual field.

### Experimental procedure

The experimental procedure was divided into two different sessions separated by a rest of 40 minutes approximately. The first session lasted 30 minutes approximately while the second was 15 minutes long. Participants wore their habitual refractive correction (either glasses or soft contact lenses) during all measurements.

The first session started by checking that the patient met the inclusion criteria of normal visual acuity and absence of strabismus. Then, the cover-uncover test was performed at 40 cm and the heterophoria was measured with a prism bar. While the patient fixated the stimulus, special emphasis was put on the importance of maintaining sharp vision in order to stimulate properly accommodation [24]. The examiner covered each patients’ eye in turn for approximately 5 seconds and increased the prism power to neutralize the recovery movement until its direction was reversed. Then, the considered value of heterophoria was the midpoint of all the range of prism powers with which no movement was perceived. A single measurement of heterophoria was obtained by placing the prism bar in front of either the right or the left eye.

After the cover-uncover test, the modified Thorington test was performed at 40 cm. As the MIM card is calibrated for the right eye, the Maddox rod was held before the right eye. The patient was asked to fixate the penlight that the examiner held in the center of the card and report through which number the vertical red line seen by the right eye crossed the horizontal axis. The measured heterophoria corresponded to the number reported by the patient.

**Fig 2:**
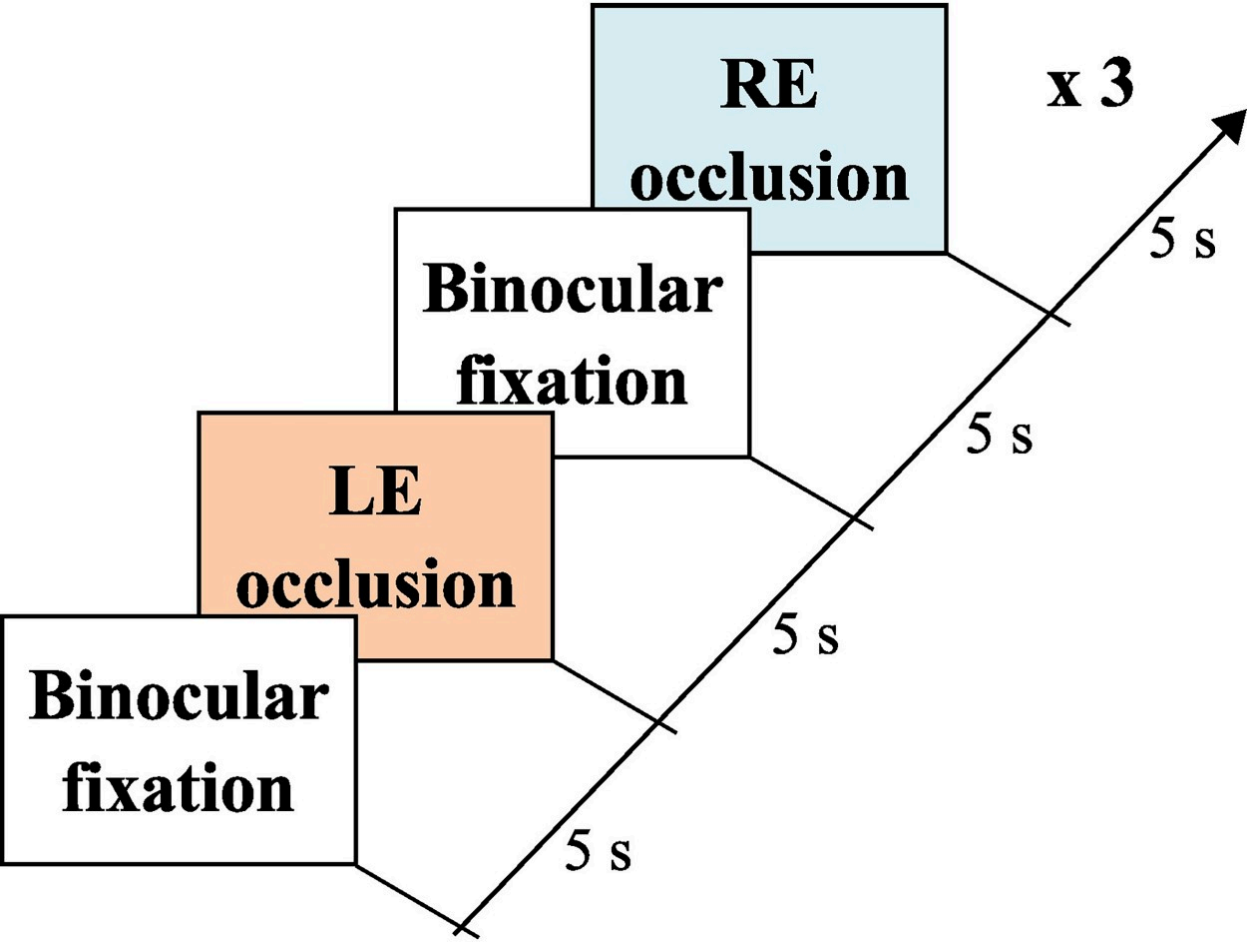
Test sequence.

Finally, the patient was positioned on a chin rest to perform the automated and objective cover test with the eye-tracker. Before starting the test, the eye-tracker was calibrated in binocular vision. Following the built-in calibration procedure of EyeLink, the patient was asked to fixate sequentially each E of the 3 x 3 grid (Fig 1B) in a non-random order. Then, the patient was asked to fixate again the same targets in order to validate the fitted model and ensure acceptable spatial accuracy. The test began immediately after the eye-tracker’s calibration. The test consisted in 3 cycles each composed of binocular fixation, left eye occlusion, binocular fixation and right eye occlusion. Each binocular or monocular fixation period lasted 5 seconds (Fig 2). Thus, the complete cover test lasted 60 seconds. The oculomotor responses during this procedure were saved for offline analysis.

This order of the tests was kept constant across all patients in order to prevent the examiner from being influenced by the response of the patient during the modified Thorington test or the movement of the eyes seen on the display of the eye-tracker’s computer during the automated and objective cover test.

The second session consisted in repeating the measurement of this automated and objective cover test for test-retest repeatability analysis. Patients were positioned in the setup and the eye-tracker was calibrated again before starting the test.

Motor ocular dominance was assessed with the Hole-in-the-Card test [25]. The test was repeated three times throughout both sessions: at the beginning and at the end of the first session and at the end of the second one. It is common to repeat the Hole-in-the-Card test several times in order to confirm motor ocular dominance [25–28]. The three answers were collapsed to two categories: “right” if the right eye was dominant the majority of times and “left” if it was the left eye.

Hereafter, we will use the initials CT, TH and ET to refer to the cover-uncover test, the modified Thorington test and the automated and objective cover test with an eye-tracker, respectively.

### Eye-tracker data processing

Binocular eye data registered with the eye-tracker were processed offline using Matlab. Periods of 100 ms before and after each blink identified by the EyeLink software were removed in order to avoid artifacts associated with the onset and offset of blinks. These empty periods were replaced by linear interpolation after confirming by visual inspection that this did not bias the traces. Additional tests were performed in preliminary analyses with a time window of 200 ms and without interpolating blink periods and results did not differ remarkably.

The eye position at each monocular or binocular period was defined as the median of the last 0.5 seconds. The displacement of the occluded eye from its previous binocular position needed to be larger than the displacement of the fixating eye in the same period in order to measure a heterophoria during that occlusion. When this condition was not fulfilled, no heterophoria was measured.

Heterophoria was computed using two different methods. Firstly, the heterophoria computed with the *1-eye* method in left eye (LE) and right eye (RE) occlusion periods, respectively, was defined as

$$Het\;LE_{1-eye}=\left(L_{occ}-L_{bin}\right)$$ (1)

$$Het\;RE_{1-eye}=-\left(R_{occ}-R_{bin}\right)$$ (2)

where *L*_*occ*_ is the position of the LE during the LE occlusion period, *L*_*bin*_ is the LE position during the binocular fixation period prior the occlusion, *R*_*occ*_ is the position of the RE during the RE occlusion period, and *R*_*bin*_ is the RE position during the binocular fixation period prior the occlusion. This method mimics the measurement conditions of the cover test, in which only the movement of the occluded eye is considered. Secondly, the heterophoria computed with the *2-eyes* method in a LE and RE occlusion periods, respectively, was defined as

$$Het\;LE_{2-eyes}=\left(L_{occ}-L_{bin}\right)+\left(R_{mon}-R_{bin}\right)$$ (3)

$$Het\;RE_{2-eyes}=-\left(R_{occ}-R_{bin}\right)-\left(L_{mon}-L_{bin}\right)$$ (4)

where *R*_*mon*_ is the position of the RE during the LE occlusion period, and *L*_*mon*_ is the position of the LE during the RE occlusion period. The deviation between the occluded *and* fixating eyes from their respective positions in the previous binocular fixation period are considered in this method. Thus, it strictly adheres to the definition which refers to heterophoria as a relative deviation between the eyes.

**Fig 3:**
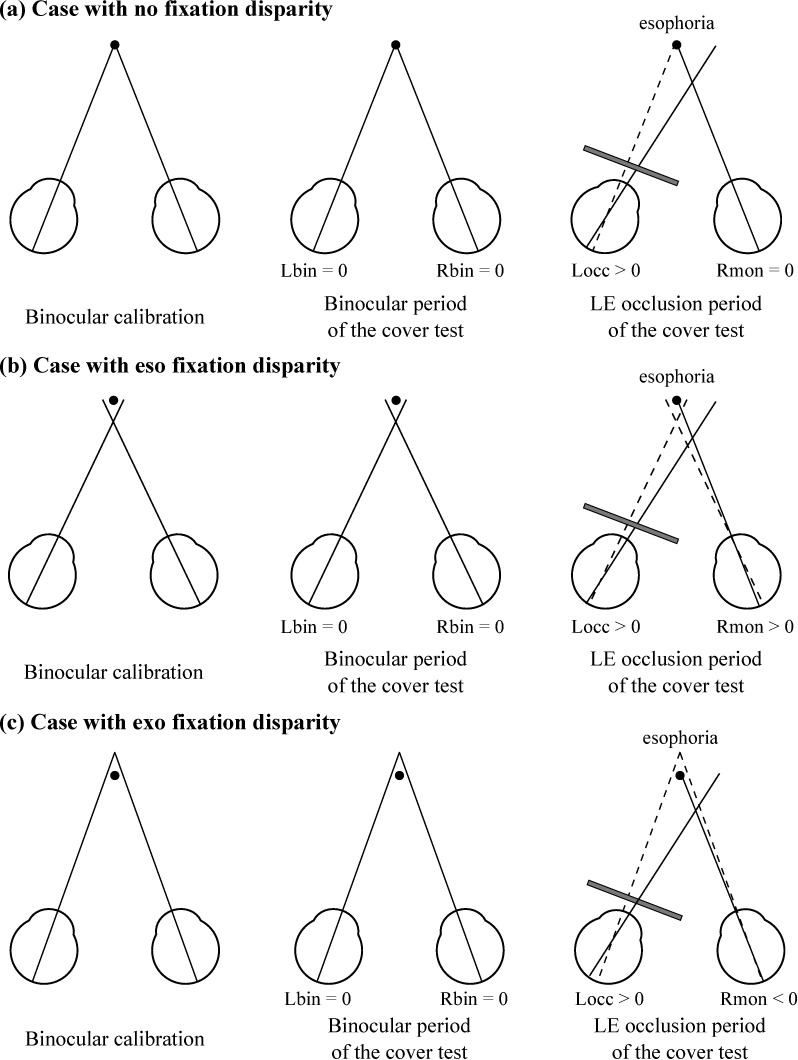
Examples of the movements of both eyes during the performance of the cover test. (a) Example of a subject with no fixation disparity. During the binocular calibration and the binocular period of the cover test both visual axes cross exactly at the target. As the subject has an esophoria, the LE turns right when it is occluded. Note that this scheme might not represent the most typical condition in which zero fixation disparity generally is accompanied by a small heterophoria. (b) Example of a subject with eso fixation disparity. In binocular viewing conditions the visual axes cross in front of the target. When the LE is occluded, it turns right to its heterophoric position and the RE refixates the target. This scheme illustrates the most physiologically plausible condition, in which esophoria occurs with eso fixation disparity. (c) Example of a subject with exo fixation disparity. In binocular viewing conditions the visual axes cross behind the target. When the LE is occluded, it turns right to its heterophoric position and the RE makes a leftward movement to refixate the target. Like (a), this scheme does not represent the most typical condition, as an exo fixation disparity generally is associated with an exophoria.

In Eqs (1)–(4), theoretically, *R*_*bin*_ and *L*_*bin*_ are expected to be 0, as the eye-tracker’s calibration was performed binocularly. The terms (*R*_*mon*_−*R*_*bin*_) and −(*L*_*mon*_−*L*_*bin*_) in Eqs (3) and (4) correspond to the RE and LE components of fixation disparity, respectively, and are added to the measured heterophoria if they are in the same direction, whereas they are subtracted from the heterophoria if the uncovered eye moves in the opposite direction of the occluded eye. Following the sign convention typically used, exophorias are negative and esophorias are positive. In both methods the final heterophoria was computed as the median of the measurements obtained across the six occlusions. Fig 3 shows examples of the movements of the occluded and fixating eyes during the cover test in subjects with and without fixation disparity.

### Statistical analysis

Statistical analyses were performed using SPSS 24 (IBM Corp., Armonk, New York, USA). The significance level was set at 0.05. The Shapiro-Wilk test was used to verify that each variable was normally distributed.

According to the sign convention typically used, exophorias were represented with a negative sign and esophorias with a positive sign. Comparing the signed heterophoria values we can know whether one condition is biased towards more esophoric or exophoric values compared to another. However, differences towards eso- and exo- direction cancel out. In order to know whether there is an over- or under-estimation of the magnitude of heterophoria in one condition compared to another regardless of its direction, the absolute values of heterophoria were also compared.

Paired t-tests were performed to determine whether the heterophoria measured with the *1-eye* and *2-eyes* methods differed significantly, to assess the intersession repeatability of the ET, and to analyze the differences in heterophoria between the eyes. A repeated measures ANOVA was used to determine the agreement between the signed heterophoria results obtained with the different methods. As our data violated the assumption of sphericity according to the Mauchly test, a Greenhouse-Geisser correction was applied. The agreement between the absolute heterophoria results of the different tests was determined with the non-parametric Friedman test since the data was not normally distributed. Post-hoc analysis with Wilcoxon signed-rank tests with Bonferroni correction was conducted. In this case, the Bonferroni correction set the significance level at *p* < 0.017 (0.05/3 = 0.017). The repeatability and agreement between CT, TH and ET were also assessed with Bland and Altman analysis [29]. Pearson’s correlation coefficients were obtained to determine the strength of association between: (1) the mean magnitude of heterophoria computed with the *1-eye* and *2-eyes* methods and the difference between them, (2) the results of heterophoria measured in the first and second sessions and (3) the difference between the heterophoria measured with the three different tests and its mean magnitude (three pairs). The Chi-square test of association (or Pearson’s Chi-square test) was used to analyze the relationship between the direction of heterophoria measured with the different methods. Finally, the Welch test was performed to determine a potential effect of motor ocular dominance on the symmetry of heterophoria between the eyes after verifying that homogeneity of variances could not be assumed according to the Levene’s test.

## Results

### Differences between *1-eye* and *2-eyes* methods

**Fig 4:**
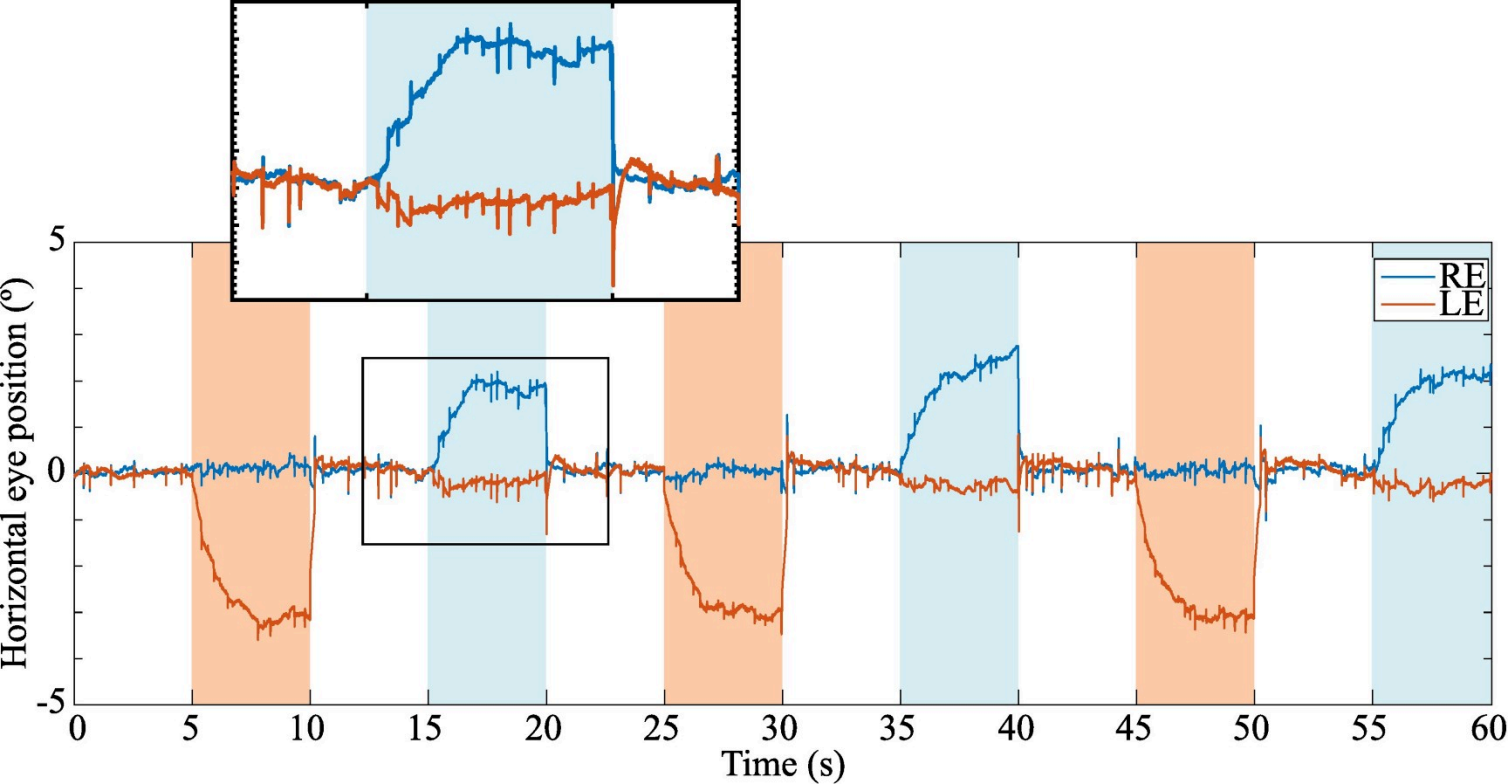
Ocular traces from a representative observer during the performance of the cover test. Horizontal RE and LE positions are represented with blue and orange lines, respectively. Periods of LE occlusion are shaded in orange and periods of RE occlusion are shaded in blue. The non-shaded areas correspond to binocular fixation periods. The inset panel zooms in on the eye traces during a RE occlusion period and shows how the RE stabilizes on its heterophoric position.

Eye movements were registered with the EyeLink 1000 Plus during the performance of the ET. Fig 4 shows an example of the ocular traces from a representative participant. It can be clearly seen how both eyes point to the fixation target during the binocular periods (their horizontal position is around 0°) and how one eye deviates to reach its heterophoric position when it is occluded. The accuracy of the eye-tracker reported by the calibration step performed immediately before the test was 0.27° ± 0.11° for both RE and LE averaged across patients and sessions.

**Fig 5:**
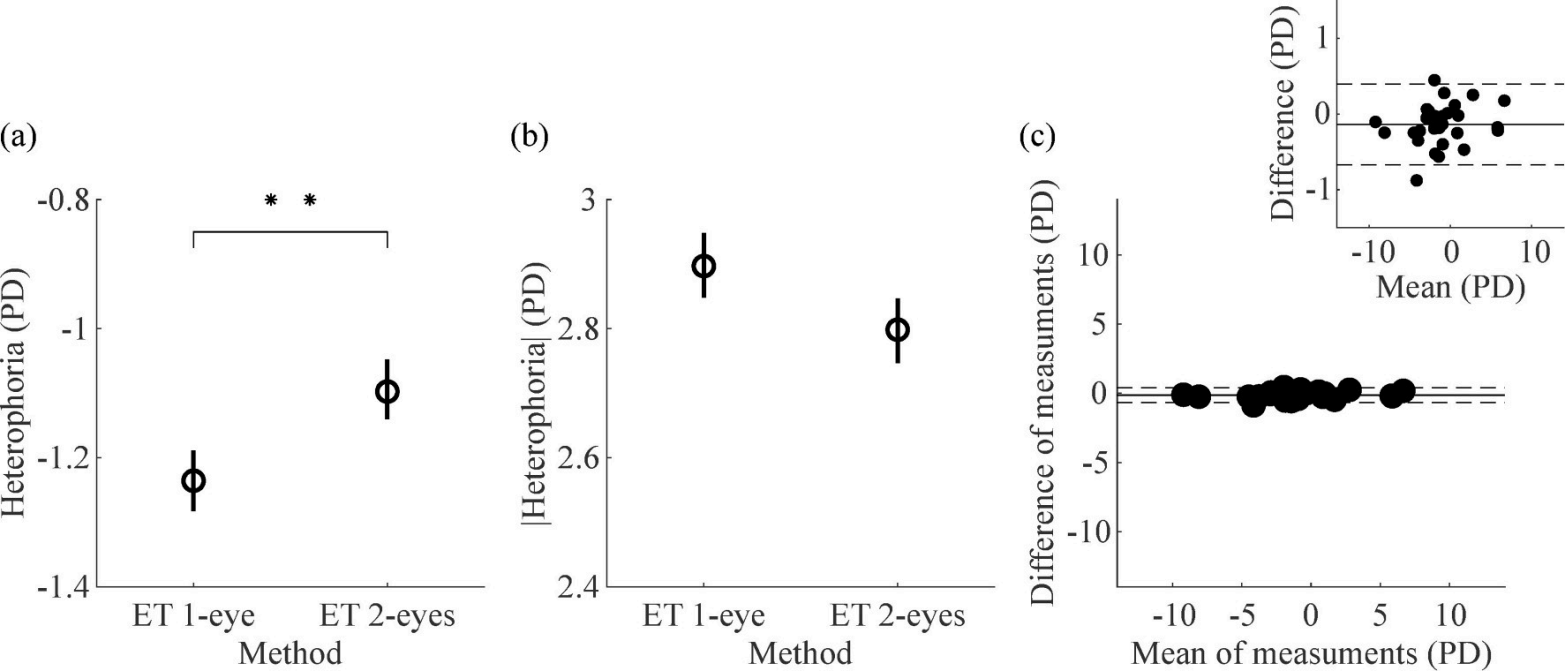
Agreement between ET heterophoria measurements obtained with the *1-eye* and *2-eyes* methods. (a) Heterophoria measurements obtained with the *1-eye* and *2-eyes* methods, averaged across sessions and participants. ** p<0.01 (b) Magnitude (absolute value) of heterophoria measurements obtained with the *1-eye* and *2-eyes* methods, averaged across sessions and participants. In (a,b) circles are means and error bars represent 95% within-subject bootstrapped confidence intervals of the mean. (c) Bland and Altman plot showing the differences between *1-eye* and *2-eyes* ET heterophoria measurements as a function of the mean of both methods. The solid line represents the mean difference between methods. The dashed lines show the 95% limits of agreement. The inset in (c) shows the same plot, rescaled along the y-axis.

The mean ± SD heterophoria averaged across participants and sessions was -1.24 ± 3.53 PD measured with the *1-eye* method, and -1.10 ± 3.47 PD measured with the *2-eyes* method (Fig 5A). The *1-eye* method results were significantly biased towards more negative, exophoric values than those produced by the *2-eyes* method [t(29) = -2.79, *p* = 0.009].

The mean ± SD heterophoria in absolute value (i.e. the magnitude of the deviation independent of whether subjects exhibited exo- or esophorias) was 2.90 ± 2.32 PD measured with the *1-eye* method, and 2.80 ± 2.28 PD measured with the *2-eyes* method (Fig 5B). There were no significant differences between the magnitude of heterophoria measured with the *1-eye* and *2-eyes* methods [t(29) = 1.88, *p* = 0.071].

In spite of the statistically significant differences between ET heterophoria measurements computed with the *1-eye* and *2-eyes* methods, we nevertheless observed a good level of agreement between the two methods. The Bland-Altman plot in Fig 5C shows how the range of observed differences between *1-eye* and *2-eyes* ET heterophoria measurements is a small fraction of the range of observed measurements averaged across the two methods. The inset in Fig 5C shows that there was not a significant correlation between the mean of the two measures and the difference between them (*r* = 0.21, *p* = 0.258).

Hereafter, the reported results of ET will correspond to the heterophoria values computed with the *2-eyes* method since it keeps strictly to the theoretical definition of this latent deviation of the visual axes.

### Repeatability of ET method

The repeatability of the ET heterophoria was determined within and between sessions. Intrasession repeatability was assessed considering the six measurements done consecutively at each occlusion. In the first session, the direction of the deviation was consistent across all occlusions except for four subjects whose magnitude of heterophoria did not exceed 1 PD. The within-subjects standard deviation of the six heterophoria measurements was 1.11 PD. Considering the ET data obtained in the second session, the within-subjects standard deviation of the six partial measurements was 0.95 PD. In this case the direction of the deviation also agreed across all occlusions except for four subjects.

**Fig 6:**
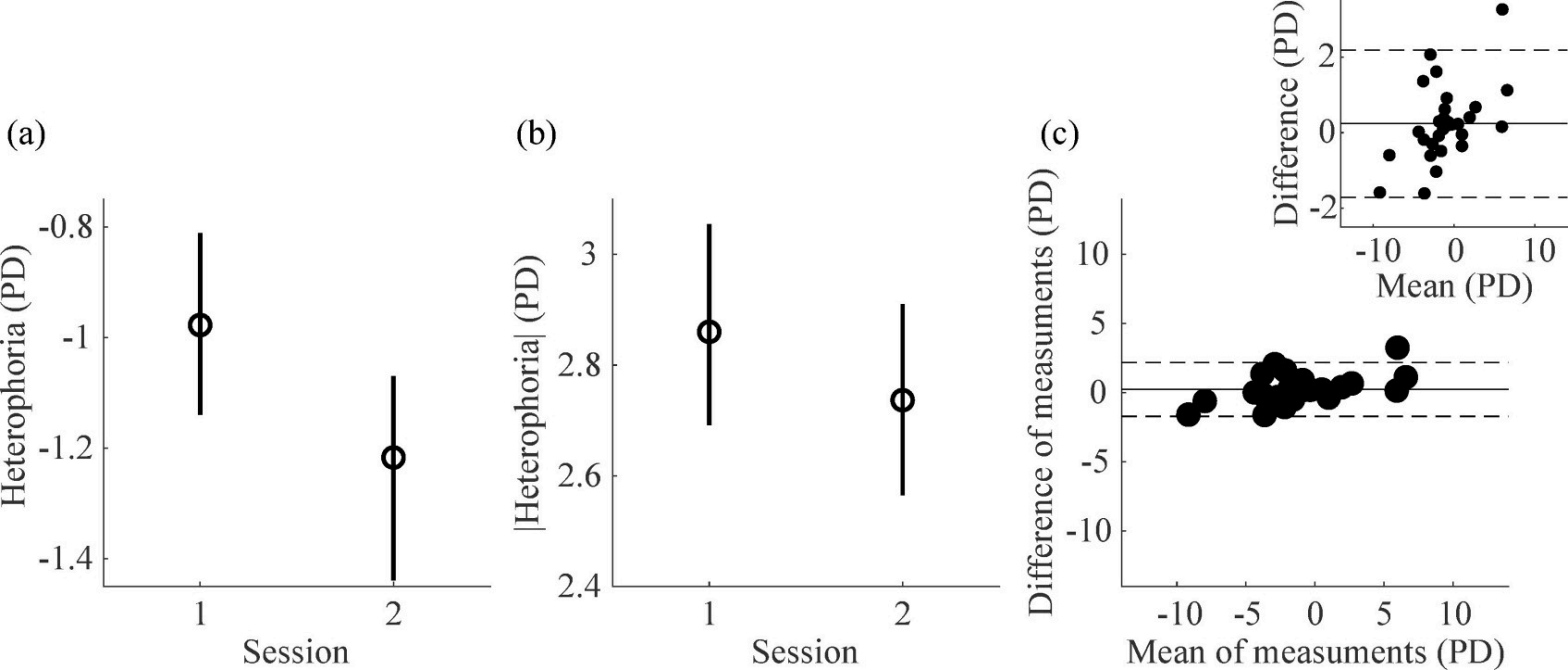
Repeatability of the ET method. (a) Heterophoria measurements obtained with the ET 2-eyes method in the first and second sessions, averaged across participants. (b) Magnitude (absolute value) of ET 2-eyes heterophoria measurements obtained in the first and second sessions, averaged across participants. In (a,b) circles are means and error bars represent 95% within-subject bootstrapped confidence intervals of the mean. (c) Bland and Altman plot showing the differences between the two sessions as a function of the mean of them. The solid line represents the mean difference between methods. The dashed lines show the 95% limits of agreement. The inset in (c) shows the same plot rescaled along the y-axis.

Intersession repeatability compares the results of the ET heterophoria of the two different sessions. The direction of the deviation was the same between sessions in all subjects. On average, the level of heterophoria measured with the ET method in the first session was -0.98 ± 3.75 PD. It did not differ significantly from the mean ± SD heterophoria measured in the second session, that was -1.22 ± 3.25 PD [t(29) = -1.32, *p* = 0.197] (Fig 6A). This implies that there was no bias towards more exo- or esophoric values between the two sessions. The two measurements were highly correlated (*r* = 0.97, *p*<0.001). The within-subjects standard deviation of the two sessions was 0.71 PD.

Considering the absolute values, the mean ± SD heterophoria measured with the ET method was 2.86 ± 2.57 PD in the first session and 2.74 ± 2.08 PD (Fig 6B). There was no tendency towards an increment or a decrease of the magnitude of heterophoria measured with the ET method between the two sessions [t(29) = 0.67, *p* = 0.508].

The Bland-Altman plot in Fig 6C shows the high intersession repeatability results, with a small mean difference and 95% limits of agreement of ±1.95 PD. In most participants the difference of heterophoria between the two sessions was smaller than what it is typically considered as clinically different.

### Agreement between CT, TH and ET methods

**Fig 7:**
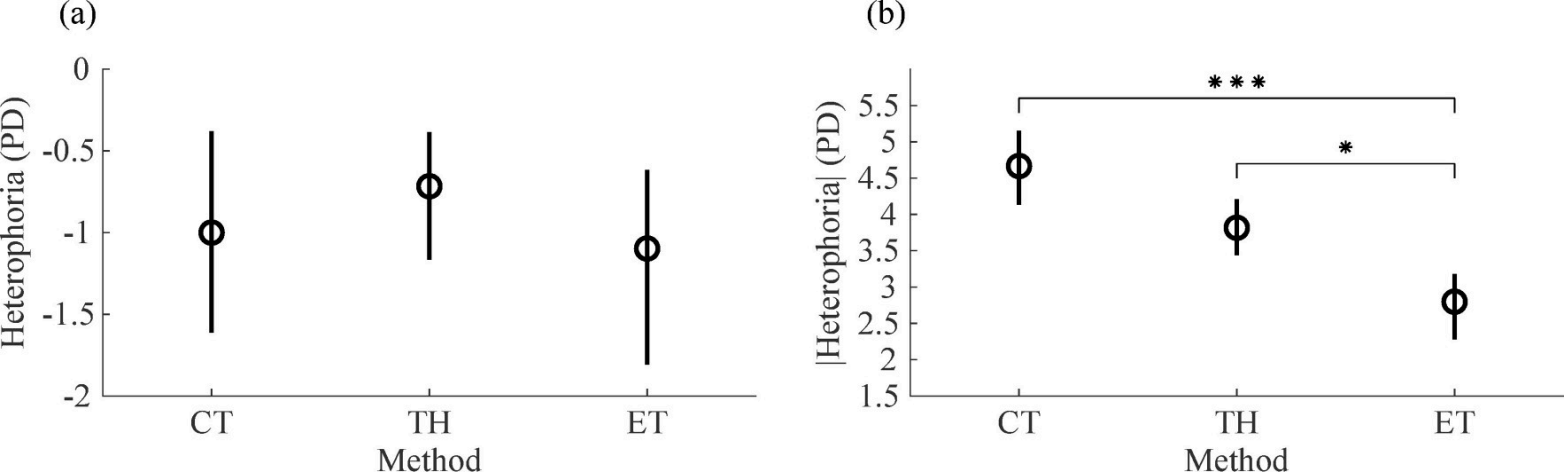
Agreement between CT, TH and ET methods. (a) Heterophoria measurements obtained with the CT, TH and ET methods. CT and TH results are averaged across participants and ET results are averaged across sessions and participants. (b) Magnitude (absolute value) of heterophoria measurements obtained with the CT, TH and ET methods, averaged across participants for the CT and TH, and across sessions and participants for the ET. Circles are means and error bars represent 95% within-subject bootstrapped confidence intervals of the mean. * p<0.05; *** p<0.001.

The agreement between the three tests used to measure horizontal heterophoria was also assessed. Individually, the mean ± SD signed heterophoria was -1.00 ± 6.35 PD with CT, -0.72 ± 5.12 PD with TH and -1.10 ± 3.47 PD with ET (Fig 7A). The heterophoria results of the ET method reported here were first averaged across sessions. A repeated measures ANOVA with a Greenhouse-Geisser correction showed no statistically significant differences between the mean heterophoria measured with the three different methods [F(1.582,45.874) = 0.31, *p* = 0.683]. This implies that none of the methods were significantly biased towards more exo- or esophoric values.

Non-parametric statistical tests were used to analyze the agreement of the absolute magnitude of heterophoria measured with the three different methods. The median (interquartile range) heterophoria in absolute value was 4 PD (1.75 to 6 PD) with CT, 3.5 PD (1 to 5 PD) with TH and 2.05 PD (0.99 to 3.73 PD) with ET (Fig 7B). The Friedman test showed statistically significant differences between the magnitude of heterophoria measured with the three methods [χ^2^(2) = 14.81, *p* = 0.001]. Post hoc analysis with Wilcoxon signed-rank test was conducted with a Bonferroni correction, resulting in a significance level set at *p* < 0.017. There were no significant differences between the magnitude of heterophoria measured with the two conventional clinical methods [Z = 2.217, *p* = 0.027]. However, the results of the ET method were significantly smaller than those obtained with the CT [Z = 3.569, *p*<0.001] and with the TH [Z = 2.499, *p* = 0.012] independent of whether subjects exhibited exo- or esophoria.

**Fig 8:**
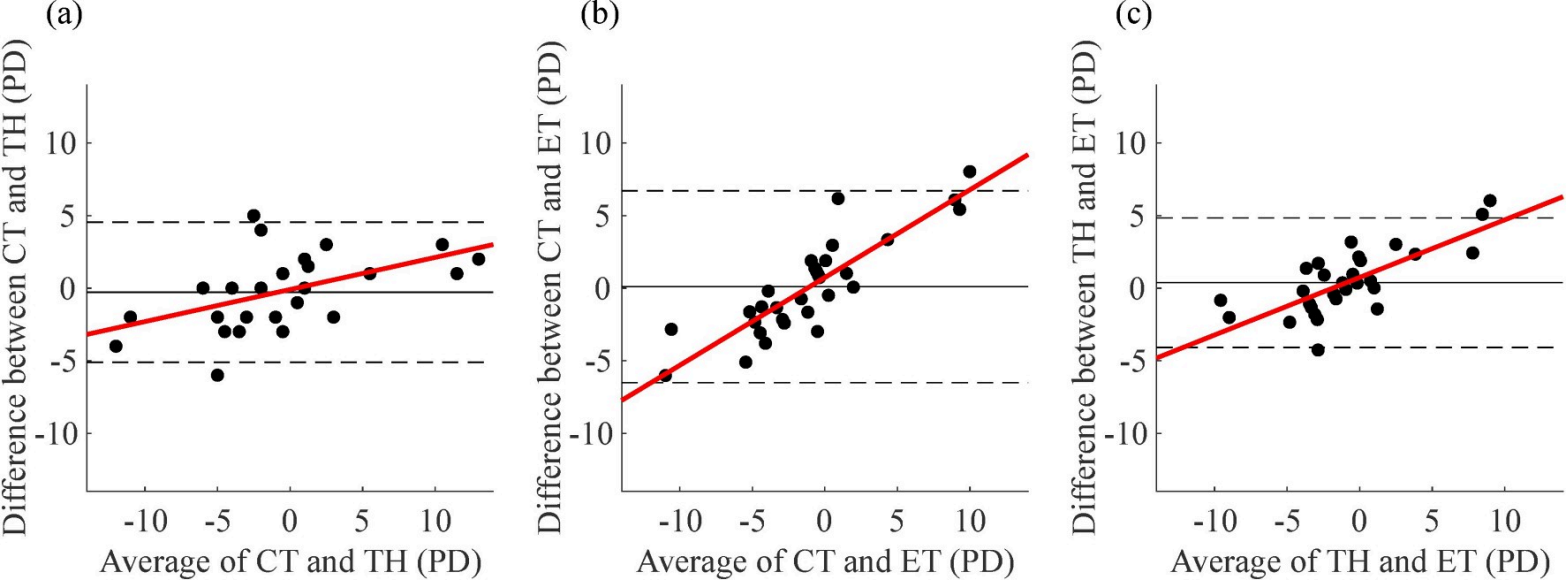
Agreement between the three pairs of methods. Bland and Altman plots comparing the CT with the TH (a), the CT with the ET (b), and the TH with the ET (c). The solid lines represent the mean difference between methods. The dashed lines show the 95% limits of agreement. The best fitting regression line through each pair of data is shown in red.

The Bland-Altman plots in Fig 8 show a mean difference between methods close to 0 PD in the three pairs although wide 95% limits of agreement. The ET method showed narrower 95% limits of agreement with the TH than with the CT. Additionally, the plots in Fig 8 show a tendency towards poorer agreement for larger magnitudes of heterophoria, either exophoria or esophoria. The Pearson’s correlation coefficient between the differences of the two measures and the average of them was 0.51 (*p* = 0.004) for the CT–TH pair, 0.87 (*p*<0.001) for the CT–ET pair and 0.74 (*p*<0.001) for the TH–ET pair.

In order to analyze the agreement in the direction of the deviation assessed with the three different methods, all deviations smaller than 1 PD have been classified as orthophoria. This consideration is especially relevant for the ET method, in which very small deviations could be measured. There was agreement in terms of the direction of the heterophoria in 66.7% of the cases between the CT and the TH methods, in 70% of the cases between the CT and the ET methods and in 70% of the cases between the TH and the ET methods. The association between the percentage of exophoric (≥ 1 PD of exophoria), esophoric (≥ 1 PD of esophoria) and orthophoric (less than 1 PD of horizontal deviation) patients measured across the different methods was tested with the Chi-square test of association. It showed a statistically significant association between all pairwise comparisons: χ^2^(4) = 18.23, *p* = 0.001 for the CT–TH pair; χ^2^(4) = 19.77, *p* = 0.001 for the CT–ET pair; and χ^2^(4) = 17.55, *p* = 0.002 for the TH–ET pair.

### Effect of motor ocular dominance on heterophoria

ET heterophoria was also computed separately for each eye. Then, RE and LE heterophorias were calculated as the median of the three values of heterophoria obtained when the RE and LE were occluded, respectively.

The direction of the deviation agreed between the eyes in 27 (90%) subjects. The three subjects who showed esophoria in the RE and exophoria in the LE or vice versa were excluded from this analysis. From the other 27 subjects, 23 gave consistent answers across the three repetitions of the Hole-in-the-Card test. As a result, 63% were RE dominant and 37% were LE dominant.

**Fig 9:**
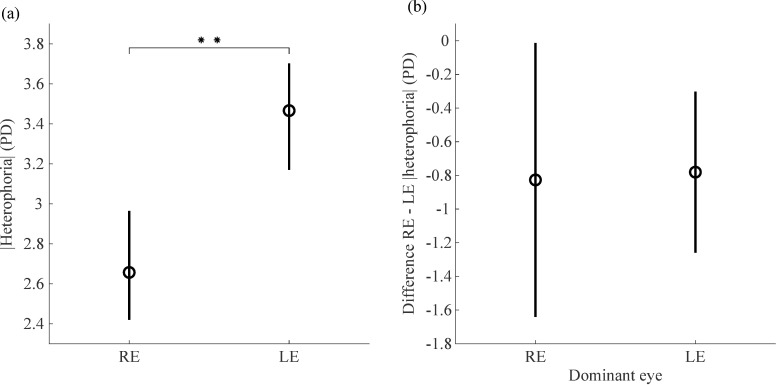
Difference between the heterophoria measured in the right and left eyes as a function of motor ocular dominance. (a) Magnitude (absolute value) of heterophoria measured separately in the right and left eyes with the ET 2-eyes method, averaged across sessions and participants. Circles are means and error bars represent 95% within-subject bootstrapped confidence intervals of the mean. ** p<0.01 (b) Difference between the magnitudes of heterophoria measured in the RE and in the LE as a function of motor ocular dominance. Circles are means and error bars represent 95% confidence intervals of the mean.

On average, the magnitude of RE heterophoria was 2.66 ± 2.35 PD (mean ± SD). The mean ± SD LE heterophoria was 3.47 ± 2.43 PD (Fig 9A). The magnitude of heterophoria in the RE was significantly smaller than in the LE [t(26) = -2.97, *p* = 0.006]. There was no significant effect of motor ocular dominance on the differences of the magnitude of heterophoria between the eyes as shown by the Welch test [t(23.901) = -0.10, *p* = 0.922] after checking that homogeneity of variances could not be assumed according to the Levene’s test (*p* = 0.03). On average, the inter-eye difference was -0.83 ± 1.71 PD for the 17 subjects with RE dominance and -0.78 ± 0.77 PD for the 10 subjects with LE dominance (Fig 9B).

## Discussion

### Differences between *1-eye* and *2-eyes* methods

According to the static position of the eyes before and after occlusion, the movements during the cover test are considered to be asymmetric vergence movements in which the fixating eye does not move when the other eye is covered. Considering the actual trajectory of the eyes, the movements are a combination of vergence an saccades in both the occluded and fixating eyes [14]. Thus, Griffin reported an occasional flick of the fixating eye in the same direction as the movement of the covered eye [22]. This movement might imply an overestimation of the magnitude of heterophoria measured with the prism cover test.

Our eye tracking recordings revealed that the fixating eye drifted slowly during the cover phase instead of maintaining steady fixation, especially in cases with large heterophoria. This drift is hypothesized to be an expression of the Hering’s law of equal innervation, which states that corresponding muscles of each eye receive equal innervations to contract or relax and perform an eye movement. For example, when a neural impulse for the performance of a rightward movement is sent out, the right lateral rectus and left medial rectus muscles receive equal innervation to contract. Another source of movement of the uncovered eye is fixation disparity. During the binocular fixation periods, the right and left lines of sight may cross behind or in front of the fixation target depending on the fixation disparity of the observer. However, when one eye is covered, the fixating eye moves to achieve precise foveal fixation and eliminate a monocular component of fixation disparity. The present discussion will be confined to heterophoria since it is generally agreed that the best practice in fixation disparity studies is to apply monocular calibrations with targets that optimize the eye tracking accuracy [21].

The impact of the movement of the fixating eye on the measurement of heterophoria might not be clinically relevant. The mean difference between the magnitudes of heterophoria measured with the *1-eye* and *2-eyes* methods, which is not artificially minimized by the positive and negative values according to the direction of the deviation, did not exceed 1.0 PD.

**Fig 10:**
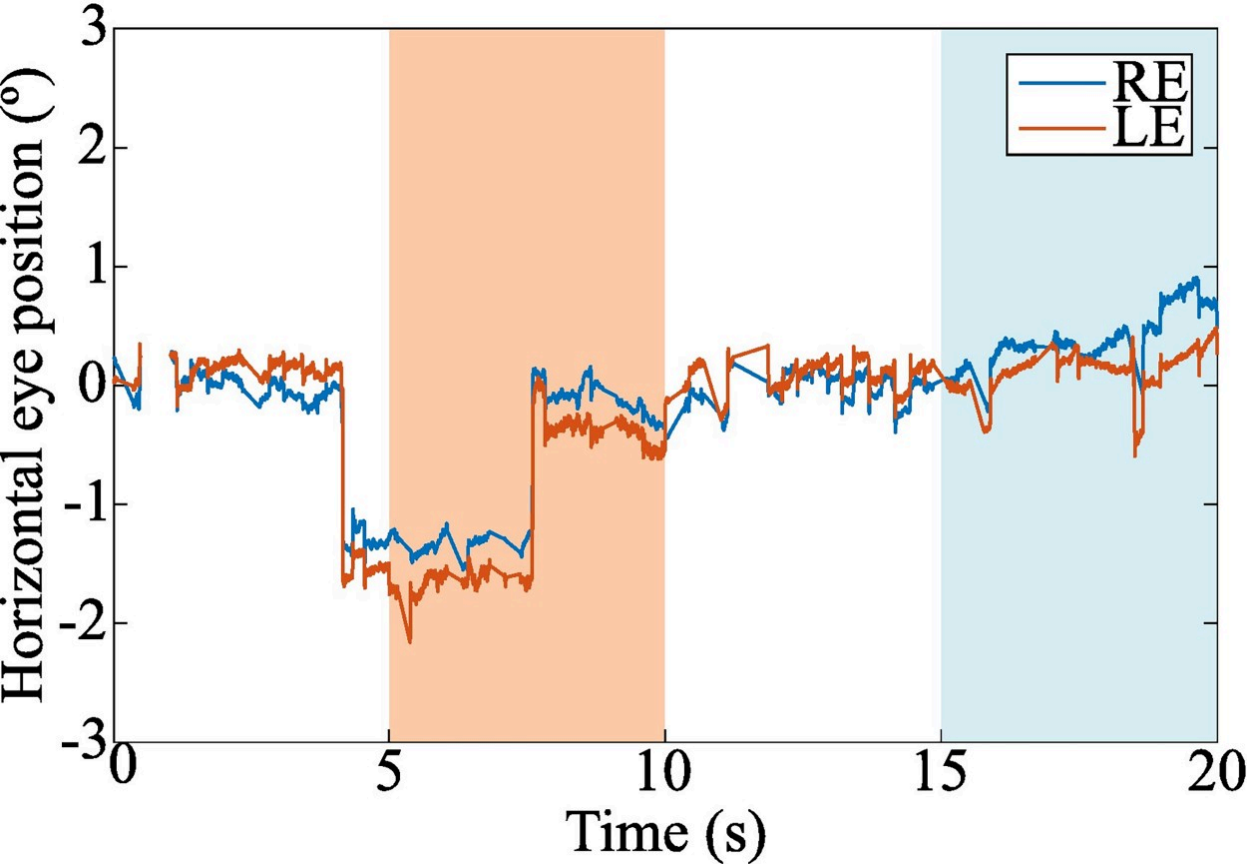
Ocular traces of a non-cooperative patient during two occlusion periods. Horizontal RE and LE positions are represented with blue and orange lines, respectively. The LE occlusion period is shaded in orange and the RE occlusion period is shaded in blue. The non-shaded areas correspond to binocular fixation periods. In the LE occlusion, a 1.80 PD esotropia of the RE would have been measured with the 1-eye method although the displacement of the RE is due to a leftward saccadic movement of both eyes.

Objective and automated measurement systems as the one used in this study need to be robust to non-desirable situations in which patients do not cooperate properly. The measurement of the relative deviation between both eyes, i.e. the *2-eyes* method, is useful to distinguish a heterophoric deviation from a conjugate movement of both eyes, e.g. a saccade. Otherwise, a saccadic movement could be measured as a heterotropia (Fig 10). In relation to that, a limitation of the method is its inability to detect and/or measure properly paralytic heterotropias, since in these conditions the secondary deviation (the deviation when the paretic eye is fixating) is always greater than the primary deviation (when the non-paretic eye is fixating) [1].

### Repeatability of ET method

**Fig 11:**
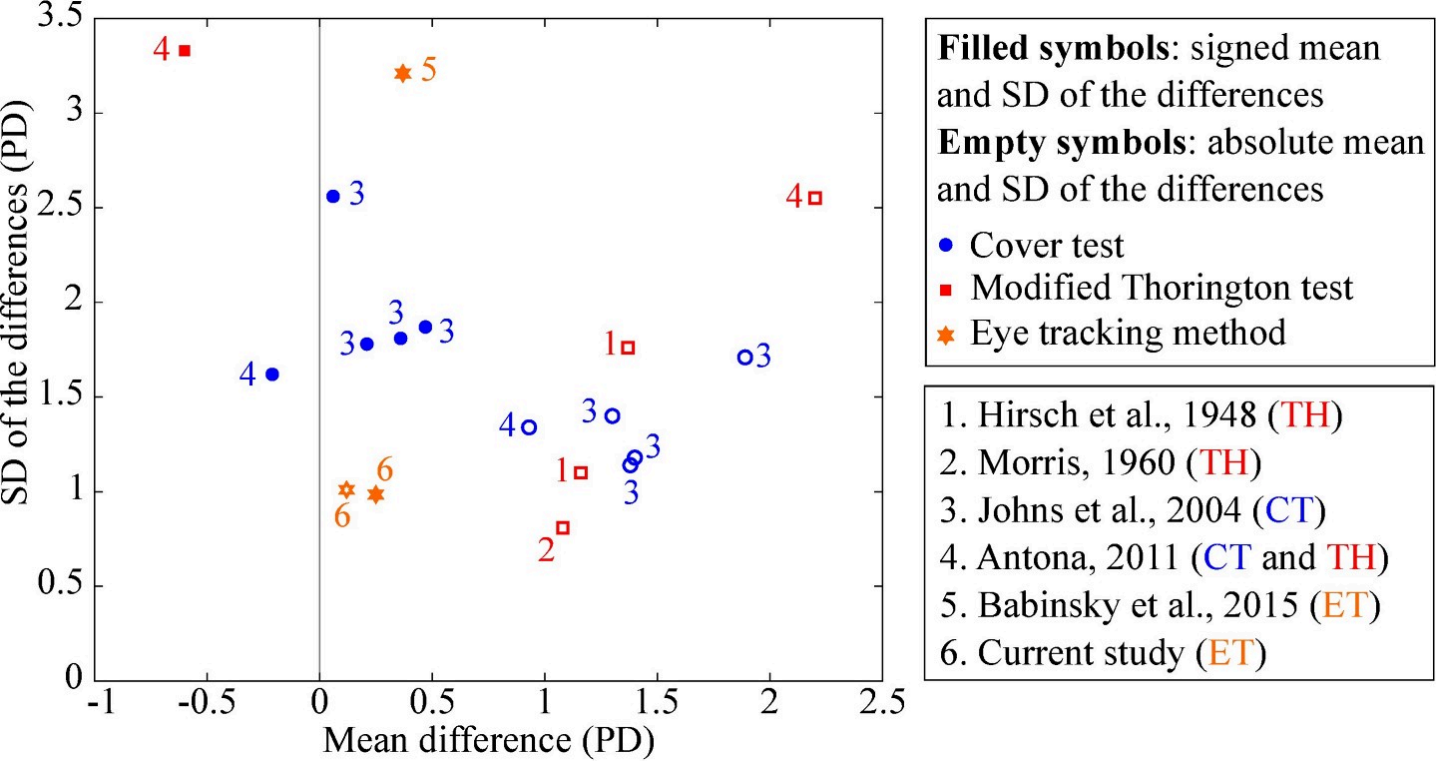
Comparison of published intersession repeatability results of different methods. Repeatability results of cover test, modified Thorington test, and eye tracking methods are represented with blue circles, red squares, and orange stars, respectively. Mean differences are plotted against the SD of the differences. Filled symbols correspond to signed mean and SD of the differences and empty symbols correspond to absolute mean and SD of the differences. Data extracted from [3,7,16,30,31].

The obtained intersession repeatability for the ET method is considerably better than the ones previously reported for the cover test and the modified Thorington test in terms of both signed and absolute mean difference (Fig 11). The articles reviewed in Fig 11 analyzed the intersession repeatability of either the cover test, the modified Thorington test, or a method to quantify heterophoria from eye tracking traces at near distance, and reported the signed and/or absolute mean difference ± SD between sessions. There are some methodological differences between studies, e.g. the sample size, the age of participants, the range of measured heterophorias or the experimental procedure, which might explain certain variability of the results.

Neither the signed nor the absolute mean difference between sessions is greater than 2 PD except for the modified Thorington test in the study from Antona (2011) [7]. This is why in general the cover test and the modified Thorington test are considered repeatable methods to measure heterophoria [3,7,30,31] according to the threshold of differences greater than 2 PD for clinical significance [11,12]. Nevertheless, the ET method proposed in the current study has the lowest mean difference between sessions and is one of the only two methods whose 95% limits of agreement (1.96 times the SD of the differences) did not exceed ±2 PD.

Intrasession repeatability computed as the within-subjects standard deviation of the six individual measures is an estimate of the precision of the final value of ET heterophoria. It is not commonly assessed for the other clinical methods since none of them is objective. Thus, consecutive measurements would be influenced by the previous responses of the patients or biased due to non-masked examiners. Alternatively, interexaminer repeatability is typically assessed. The signed mean differences between examiners ranged between 0.05 PD and 0.74 PD with SD between 1.6 PD and 2.24 PD for the cover test [3,9] and depends on the experience of the examiner [4,5]. Similar interexaminer repeatability results were obtained for the modified Thorington test [9,32].

The high intrasession and intersession repeatability results obtained with the ET method might be justified by the fact that the sources of variability are minimized. The results were not biased by the patient nor the examiner, the test was always executed equally and the experimental conditions were maintained. Thus, the found variability is likely due to physiologic variations of vergence system only.

### Agreement between CT, TH and ET methods

The signed mean differences between the heterophoria measured with the three tested methods were considerably close to 0 PD, which means that on average none of the methods were clearly biased towards more esophoric or exophoric values. To our knowledge, this is the first study that analyzes the agreement of the modified Thorington test with an eye tracking method to measure near heterophoria.

The agreement obtained in the current study between the cover test and the modified Thorington test (-0.28 ± 2.46 PD) is comparable to previous reports. Antona found a bias of the modified Thorington test towards more exophoric values with respect to the cover test with a mean difference of -0.59 PD, and 95% limits of agreement of ±5.82 PD [7]. Sanker et al. obtained a mean difference between both tests of -0.88 PD and 95% limits of agreement of ±3.64 PD [6].

Tests with similar dissociation methods and accommodative control are expected to show better agreement. Therefore, the ET method should show better agreement with the CT than with the TH. Although the mean difference between the CT–ET pair was slightly lower than between the TH–ET pair (0.10 PD and 0.38 PD, respectively), the former showed wider 95% limits of agreement (±6.62 PD and ±4.46 PD, respectively). Three methodological differences between both cover tests could justify the lack of agreement. First, it has been shown that the magnitude of heterophoria increases with the dissociation time [4,10] since it takes at least 5 seconds (and sometimes longer) for the occluded eye to reach its heterophoric position. Although the examiner attempted to cover each eye for approximately 5 seconds and left some periods of binocular fixation between occlusions in order to mimic the occlusion sequence of the ET, the number of occlusions and hence the total dissociation time was not controlled in the CT.

Besides fusional vergence, the vergence system has three more components [33]. Accommodative vergence is driven by blur, and is the consequence of the relationship between the accommodative and vergence systems [1]. Proximal vergence is driven by the perception of apparent nearness of an object. The perception of a near object stimulates both the accommodative and the vergence systems [33]. Finally, the tonic vergence is driven by the baseline neural innervation. Heterophoria depends on tonic, accommodative and proximal vergence response [24]. In particular, North et al. showed that proximal cues have a significant effect on heterophoria [34]. The second methodological difference that could justify the lack of agreement between the CT and the ET is related to proximal vergence. While tonic and accommodative vergence were stimulated similarly in the CT and the ET, the stimulation of the proximal component differed due to some particularities of the experimental setup. In both methods the test was placed at 40 cm from the patient, but in the ET a structure to support the occluders and the two motors were positioned around the patients’ face during the test. As a result, we could expect an increment in proximal vergence stimulation. One would expect in turn an increase in esophoria and/or a decrease in exophoria due to proximal effects. However, our results showed a decrease in the magnitude of heterophoria (either esophoria or exophoria) with the ET method.

Third, different criteria about the endpoint of the cover test might result in different values of heterophoria [3]. The endpoint criteria used in the prism cover test was the midpoint from reversal, while the heterophoric position registered with the eye-tracker possibly corresponds to the first neutral endpoint. Johns et al. (2004) concluded that the differences between the first neutral and the midpoint from reversal endpoints were not clinically significant. However, it is relevant to note that there was a tendency of increased differences between the two endpoints with the magnitude of heterophoria according to the reported Bland and Altman plots [3]. Therefore, the poorer agreement showed in the cases of large heterophoria might be partially due to the different endpoints used in each method together with the greater movement of the fixating eye not considered in the CT as reported above.

Another aspect that might partially justify the poor agreement between methods relies on whether a single heterophoria measurement was obtained for one eye (as in the CT and TH methods) or if it was computed as the median of the measures obtained for left and right eyes (as in the ET method). This might play a significant role in this study due to the asymmetry of heterophoria between the eyes found with the ET method. Nevertheless, the effect of the different dissociation methods between the TH and the ET might be more prominent since a similar level of agreement was obtained between the TH results and the ET heterophoria computed separately for the RE (mean difference ± SD of 0.10 ± 2.50 PD). The same analysis cannot be done with the CT-ET pair since in the CT the heterophoria was measured either on the right or the left eye without distinction.

Hrynchak et al. analyzed the agreement of the heterophoria measured with a prism bar during the alternate cover test and with a head mounted eye-tracker [5]. They obtained considerably better results, especially at the 95% limits of agreement. They considered only the deviation of the occluded eye to measure heterophoria from the eye tracking traces and did not find an effect of the deviation magnitude on the cover tests agreement. The 95% limits of agreement obtained in the current study were comparable with the ones obtained by Babinsky et al. on a sample of young children [16]. Recently, Troyer et al. obtained slightly better agreement than Babinsky yet still comparable values between cover test and objective measurements in both children and adults [35].

### Effect of motor ocular dominance on heterophoria

Regarding the symmetry between the eyes, on average heterophoria was significantly greater in the LE than in the RE. Previously, these asymmetries were attributed to the occlusion sequence and explained by the different time courses of relaxation of the fast and slow components of fusional vergence [10]. However, the results of the current study showed greater heterophoria in the first occluded eye. Similar results were obtained by van Rijn et al., who could not show an effect of the occlusion order on the asymmetry of heterophoria [36].

There was no significant effect of motor ocular dominance on the asymmetry of heterophoria between the eyes. The mean difference in heterophoria between the eyes is essentially the same in RE and LE dominant subjects. Although there is evidence for an effect of ocular dominance on the dynamics of the recovery movements [14], the amplitude of the movement during the cover phase might be independent of motor ocular dominance.

The reason for the horizontal heterophoria asymmetries found in the current study is not known. Further analyses are needed to evaluate possible causes for these differences. Particular attention was paid to center the optical axis of the camera relatively to patients’ heads and obtain symmetrically positioned elements. Asymmetries could be partially explained by differences in the accommodative state between both eyes [36]. All patients wore their habitual refractive correction during the measurements, but a monocular uncorrected refractive error might lead to a different contribution of the accommodative vergence on the magnitude of heterophoria between the eyes.

## Conclusions

The use of eye-trackers to measure heterophoria overcomes several limitations of the conventional clinical methods and offers various advantages: movements of the occluded eye can be recorded; better resolution, accuracy, and intrasession and intersession repeatability are obtained; the measure is objective; and it provides new insights into the movements of both the fixating and occluded eyes during the cover test. The possibility to have binocular and monocular recordings of the viewing and covered eye offers the opportunity to do a more complete analysis. Further studies could be done to include the measurement of objective fixation disparity and analyze its relationship with heterophoria if the eye-tracker is calibrated monocularly and with high accuracy.

None of the existing methods to measure heterophoria are interchangeable. However, as eye-trackers become common tools in clinical settings, their use should be the new gold standard for the automated and objective measurement of heterophoria.

## Supporting information

S1 Fig Experimental setup. (PDF)

S2 Fig Experimental setup from the patient’s point of view. (PDF)

S1 Dataset Spreadsheet that summarizes the heterophoria values measured with all the tested methods and the ocular dominance of all participants. (XLSX)

## References

[1] Von Noorden GK, Campos EC. Binocular Vision and Ocular Motility. 6th ed St Louis: Mosby; 2002.

[2] Scheiman M, Wick B. Clinical management of Binocular Vision. Heterophoric, Accommodative, and Eye Movement Disorders. 4th ed. Philadelphia: Lippincott Williams & Wilkins; 2014.

[3] Johns HA, Manny RE, Fern K, Hu Y-S. The intraexaminer and interexaminer repeatability of the alternate cover test using different prism neutralization endpoints. Optom Vis Sci. 2004;81(12):939–46. 15592119

[4] Anderson HA, Manny RE, Cotter SA, Mitchell GL, Irani JA. Effect of examiner experience and technique on the alternate cover test. Optom Vis Sci. 2010;87(3):168–75. 10.1097/OPX.0b013e3181d1d954 20125058 PMC3740008

[5] Hrynchak PK, Herriot C, Irving EL. Comparison of alternate cover test reliability at near in non-strabismus between experienced and novice examiners. Ophthalmic Physiol Opt. 2010;30(3):304–9. 10.1111/j.1475-1313.2010.00723.x 20444138

[6] Sanker N, Prabhu A, Ray A. A comparison of near-dissociated heterophoria tests in free space. Clin Exp Optom. 2012;95(6):638–42. 10.1111/j.1444-0938.2012.00785.x 22882343

[7] Antona B, Gonzalez E, Barrio A, Barra F, Sanchez I, Cebrian JL. Strabometry precision: intra-examiner repeatability and agreement in measuring the magnitude of the angle of latent binocular ocular deviations (heterophorias or latent strabismus). Binocul Vis Strabol Q Simms Rom. 2011;26(2):91–104. 21736550

[8] Cebrian JL, Antona B, Barrio A, Gonzalez E, Gutierrez A, Sanchez I. Repeatability of the Modified Thorington Card Used to Measure Far Heterophoria. Optom Vis Sci. 2014;91(7):786–92. 10.1097/OPX.0000000000000297 24901486

[9] Rainey BB, Schroeder TL, Goss DA, Grosvenor TP. Inter-Examiner Repeatability of Heterophoria Tests. Optom Vis Sci. 1998;75(10):719–26. 9798211 10.1097/00006324-199810000-00016

[10] Barnard NAS, Thomson D. A quantitative analysis of eye movements characteristics during the cover test—a preliminary report. Ophthalmic Physiol Opt. 1995;15(5):413–9. 8524567

[11] Ludvigh E. Amount of eye movement objetively perceptible to the unaided eye. Am J Ophthalmol. 1949;32:649–50. 18121093 10.1016/0002-9394(49)91415-4

[12] Fogt N, Baughman BJ, Good G. The effect of experience on the detection of small eye movements. Optom Vis Sci. 2000;77(12):670–4. 11147737 10.1097/00006324-200012000-00014

[13] Pickwell D. Binocular Vision Anomalies: Investigation and Treatment. Oxford: Butterworth-Heinemann; 1989. 15–21 p.

[14] Peli E, McCormack G. Dynamics of cover test eye movements. Am J Optom Physiol Opt. 1983;60(8):712–24. 6624871 10.1097/00006324-198308000-00010

[15] Han SJ, Guo Y, Granger-Donetti B, Vicci VR, Alvarez TL. Quantification of heterophoria and phoria adaptation using an automated objective system compared to clinical methods. Ophthalmic Physiol Opt. 2010;30(1):95–107. 10.1111/j.1475-1313.2009.00681.x 19682268

[16] Babinsky E, Sreenivasan V, Rowan Candy T. Near heterophoria in early childhood. Investig Ophthalmol Vis Sci. 2015;56(2):1406–15. 25634983 10.1167/iovs.14-14649 PMC4340430

[17] Jaschinski W, Jainta S, Kloke WB. Objective vs subjective measures of fixation disparity for short and long fixation periods. Ophthalmic Physiol Opt. 2010 7;30:379–90. 10.1111/j.1475-1313.2010.00753.x 20629960

[18] Jaschinski W. Individual Objective and Subjective Fixation Disparity in Near Vision. Martinez-Conde S, editor. PLoS One. 2017 1 30;12(1):e0170190 10.1371/journal.pone.0170190 28135308 PMC5279731

[19] Jaschinski W. Pupil size affects measures of eye position in video eye tracking: implications for recording vergence accuracy. J Eye Mov Res. 2016;9(4):1–14.

[20] Schroth V, Joos R, Jaschinski W. Effects of Prism Eyeglasses on Objective and Subjective Fixation Disparity. Kapoula Z, editor. PLoS One. 2015 10 2;10(10):e0138871 10.1371/journal.pone.0138871 26431525 PMC4592239

[21] Švede A, Treija E, Jaschinski W, Krūmiņa G. Monocular versus binocular calibrations in evaluating fixation disparity with a video-based eye-tracker. Perception. 2015;44:1110–28. 10.1177/0301006615596886 26562925

[22] Griffin JR. Binocular anomalies: Procedures for vision therapy. 2nd et. New York: Professional Press Books; 1982.

[23] Pickwell LD. Eye movements during the cover test. Br J Physiol Opt. 1973;28:23–5. 4792002

[24] Schroeder TL, Rainey BB, Goss DA, Grosvenor TP. Reliability of and Comparisons Among Methods of Measuring Dissociated Phoria. Optom Vis Sci. 1996;73(6):389–97. 8807650 10.1097/00006324-199606000-00006

[25] Li J, Lam CSY, Yu M, Hess RF, Chan LYL, Maehara G, et al Quantifying sensory eye dominance in the normal visual system: A new technique and insights into variation across traditional tests. Investig Ophthalmol Vis Sci. 2010;51(12):6875–81. 20610837 10.1167/iovs.10-5549

[26] Johansson J, Seimyr GÖ, Pansell T. Eye dominance in binocular viewing conditions. J Vis. 2015;15(9):1–17. 10.1167/15.9.1 26230983

[27] Zhou D, Ni N, Ni A, Chen Q, Hu D-N, Zhou J. Association of Visual Acuity with Ocular Dominance in 2045 Myopic Patients. Curr Eye Res. 2017;42(8):1155–9. 10.1080/02713683.2017.1297464 28494159

[28] Rice ML, Leske DA, Smestad CE, Holmes JM. Results of ocular dominance testing depend on assessment method. J AAPOS. 2008 8;12(4):365–9. 10.1016/j.jaapos.2008.01.017 18455935 PMC2679867

[29] Bland JM, Altman DG. Statistical Methods for Assessing Agreement Between Two Methods of Clinical Measurement. Lancet. 1986;1(8476):307–10. 2868172

[30] Hirsch MJ, Bing LB. The effect of testing method on values obtained for phoria at forty centimeters. Am J Optom Arch Am Acad Optom. 1948;25(9):407–16. 18882006 10.1097/00006324-194809000-00001

[31] Morris FM. The influence of kinesthesis upon near heterophoria measurements. Am J Optom Arch Am Acad Optom. 1960;37(7):327–51. 14424315 10.1097/00006324-196007000-00001

[32] Wong EPF, Fricke TR, Dinardo C. Interexaminer Repeatability of a New, Modified Prentice Card Compared with Established Phoria Tests. Optom Vis Sci. 2002;79(6):370–5. 12086303 10.1097/00006324-200206000-00010

[33] Ciuffreda KJ. Components of clinical near vergence testing. J Behav Optom. 1992;3(1):3–13.

[34] North R V, Henson DB, Smith TJ. Influence of proximal, accommodative and disparity stimuli upon the vergence system. Ophthalmic Physiol Opt. 1993;13:239–43. 8265164 10.1111/j.1475-1313.1993.tb00465.x

[35] Troyer ME, Sreenivasan V, Peper TJ, Candy TR. The heterophoria of 3–5 year old children as a function of viewing distance and target type. Ophthalmic Physiol Opt. 2017;37(1):7–15. 10.1111/opo.12342 27921322 PMC5195916

[36] Van Rijn LJ, Ten Tusscher MPM, De Jong I, Hendrikse F. Asymmetrical vertical phorias indicating dissociated vertical deviation in subjects with normal binocular vision. Vision Res. 1998;38(19):2973–8. 9797992 10.1016/s0042-6989(98)00079-0

