# Supplementary figures and images for: An automated and objective cover test to measure heterophoria
Source: PLoS One. 2018 Nov 1;13(11):e0206674. doi: 10.1371/journal.pone.0206674 (PMC6211723; doi:10.1371/journal.pone.0206674)

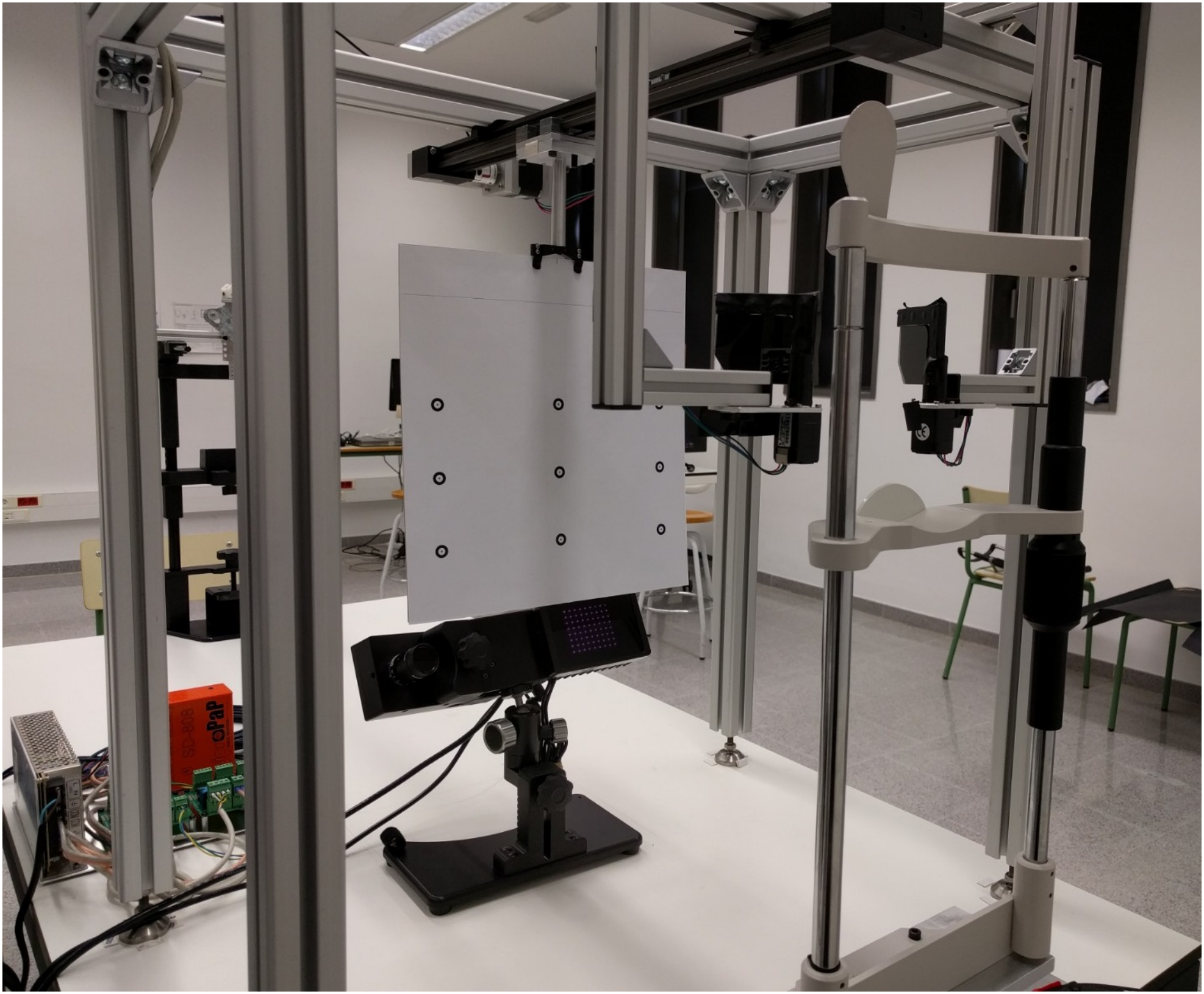

Supplement: S1 Fig — (PDF) [file pone.0206674.s001.pdf]

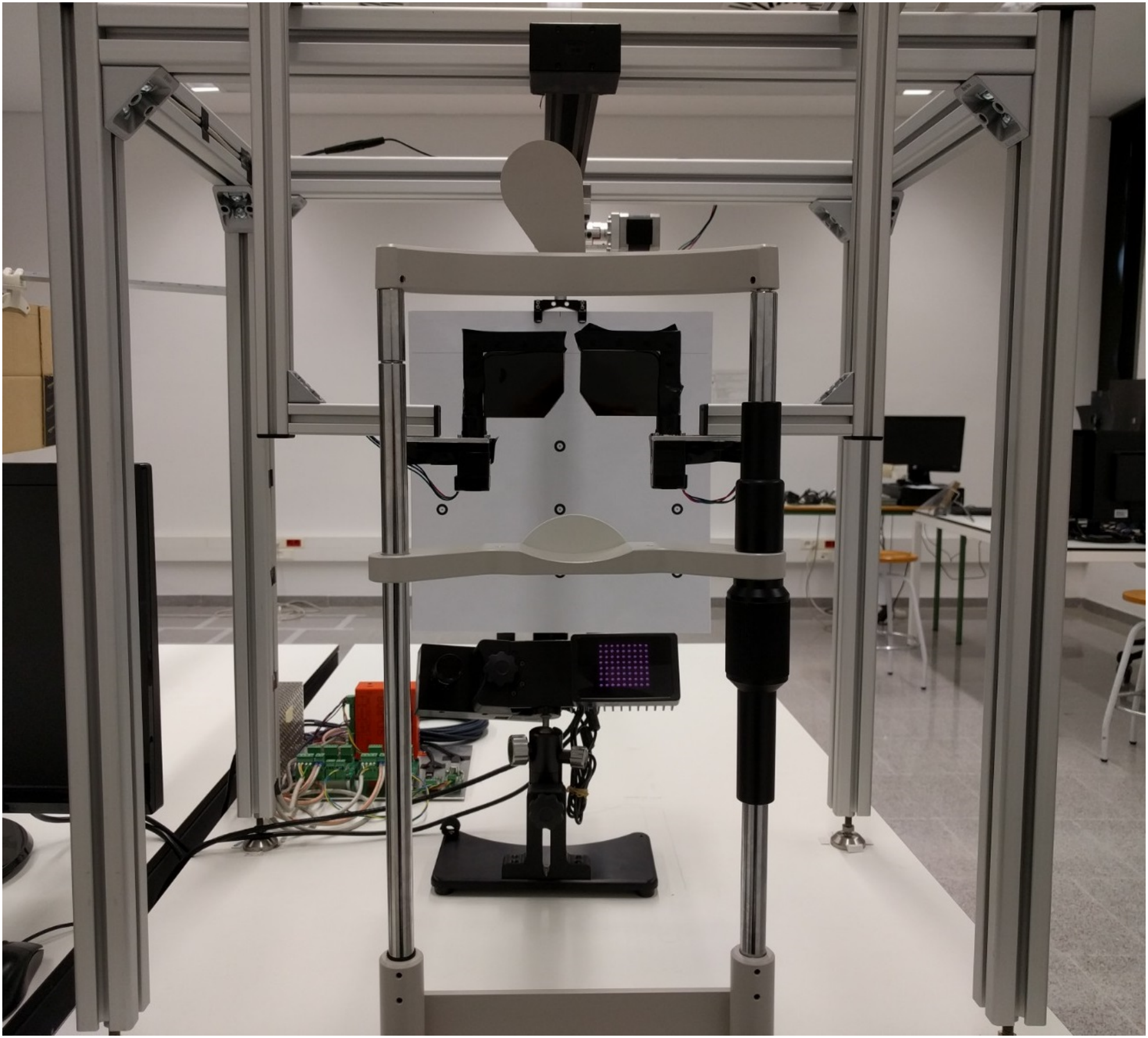

Supplement: S2 Fig — (PDF) [file pone.0206674.s002.pdf]
